# Patterns of relapse in primary central nervous system lymphoma: inferences regarding the role of the neuro-vascular unit and monoclonal antibodies in treating occult CNS disease

**DOI:** 10.1186/s12987-017-0064-3

**Published:** 2017-06-02

**Authors:** Prakash Ambady, Rongwei Fu, Joao Prola Netto, Cymon Kersch, Jenny Firkins, Nancy D. Doolittle, Edward A. Neuwelt

**Affiliations:** 1 Department of Neurology, Oregon Health & Science University, 3181 SW Sam Jackson Park Road, L603, Portland, OR 97239 USA; 2 Portland Veterans Affairs Medical Center, Portland, OR USA; 3 School of Public Health, Oregon Health & Science University, Portland, OR USA; 4 Department of Emergency Medicine, Oregon Health & Science University, Portland, OR USA; 5 Department of Radiology, Oregon Health & Science University, Portland, OR USA; 6 Department of Neurosurgery, Oregon Health & Science University, Portland, OR USA

## Abstract

**Background and purpose:**

The radiologic features and patterns of primary central nervous system lymphoma (PCNSL) at initial presentation are well described. High response rates can be achieved with first-line high-dose methotrexate (HD-MTX) based regimens, yet many relapse within 2 years of diagnosis. We describe the pattern of relapse and review the potential mechanisms involved in relapse.

**Methods:**

We identified 78 consecutive patients who attained complete radiographic response (CR) during or after first-line treatment for newly diagnosed PCNSL (CD20+, diffuse large B cell type). Patients were treated with HD-MTX based regimen in conjunction with blood–brain barrier disruption (HD-MTX/BBBD); 44 subsequently relapsed. Images and medical records of these 44 consecutive patients were retrospectively reviewed. The anatomical location of enhancing lesions at initial diagnosis and at the time of relapse were identified and compared.

**Results:**

37/44 patients fulfilled inclusion criteria and had new measureable enhancing lesions at relapse; the pattern and location of relapse of these 37 patients were identified. At relapse, the new enhancement was at a spatially distinct site in 30 of 37 patients. Local relapse was found only in seven patients.

**Discussion:**

Unlike gliomas, the majority of PCNSL had radiographic relapse at spatially distinct anatomical locations within the brain behind a previously intact neurovascular unit (NVU), and in few cases outside, the central nervous system (CNS). This may suggest either (1) reactivation of occult reservoirs behind an intact NVU in the CNS (or ocular) or (2) seeding from bone marrow or other extra CNS sites.

**Conclusion:**

Recognizing patterns of relapse is key for early detection and may provide insight into potential mechanisms of relapse as well as help develop strategies to extend duration of complete response.

## Background

Primary central nervous system lymphoma (PCNSL) in immunocompetent patients (non-acquired immune deficiency syndrome and non-post-transplant lymphoproliferative disease) is a rare, aggressive extra-nodal non-Hodgkin’s lymphoma. The most common morphology consists primarily of diffuse large CD20+ B-cell aggregates confined to the CNS or eyes at initial presentation. First line high dose methotrexate (HD-MTX)-based chemotherapy regimens are the current backbone therapy for newly diagnosed PCNSL with high rates of complete response (CR) [1]. CR is defined by the complete disappearance of all enhancing abnormalities on gadolinium-enhanced MRI with no evidence of disease in the CSF and ocular compartments after discontinuation of all corticosteroids for at least 2 weeks [2]. Despite high initial CR rates with MTX-based regimens, over 50% of patients relapse within 2 years of diagnosis [3–5]. Unlike systemic diffuse large B-cell lymphoma (DLBCL), PCNSL lack a plateau in progression-free survival rates; even patients who remain disease free for over 5 years continue to be at risk of relapse [6]. Understanding the mechanisms of relapse is particularly important to further improve overall survival by guiding therapies aimed at extending disease control [7].

Primary central nervous system lymphoma in immunocompetent patients typically presents as a solitary homogeneously enhancing mass in the subcortical white matter, predominantly in the periventricular or white matter of the cerebral hemispheres [8–10]. Contrast-enhanced MRI is the preferred imaging technique for diagnosis, response assessment and follow up. Lesions are typically hypo- or isointense on T1-weighted MR images and iso-, hypo-, or hyperintense on T2-weighted MR images with evidence of restricted diffusion [11–13]. Although the characteristic feature of newly diagnosed PCNSL in immunocompetent patients is well described, the pattern and location of relapses is not. Relapses are generally believed to be derived from the same clone as the initial presentation and not entirely new disease [14, 15]. It has been postulated that relapse may be due to seeding from occult CNS sites, ocular disease or from distant subclinical extra-CNS sites [7]. Better understanding of the pattern and mechanism of relapse is key to early detection and understanding the true extent of disease, potentially helping guide therapies aimed at maintaining response as well as better manage relapses. We report the site of relapse in PCNSL patients after attaining CR with HD-MTX in conjunction with blood–brain barrier disruption (BBBD).

## Methods

### Patients

Our institutional review board approved this study. This retrospective review identified all newly-diagnosed immunocompetent PCNSL patients treated with HD-MTX/BBBD between 02/1982 and 09/2013 at our institution. Inclusion criteria included: (1) histologically confirmed CD20+ DLBCL confined to the brain, cerebrospinal fluid or eyes; (2) treatment with intra-arterial HD-MTX/BBBD regimens with or without rituximab (treatment regimens were previously described) [16, 17]; (3) first relapse after achieving CR with first line treatment. Patients with primary low grade CNS lymphoma and primary CNS T-cell lymphoma, evidence of lymphoma outside the CNS at initial presentation, having only ocular lymphoma but subsequently developed CNS lesion before CR in the eyes, and patients with no measurable radiologic lesions (diagnosis only by CSF analysis) were excluded. Patients who received alternative therapies/regimens (other than HD-MTX/BBBD) as first line therapy, whole-brain radiotherapy (WBRT), or maintenance immunotherapy after completion of initial year of therapy, were also excluded from the analysis. Only patients with documented radiologic relapse were included, since pattern of relapses were the focus of this analysis.

### Radiologic assessment

Imaging and response assessment was done as previously described and in line with current international consensus-based guidelines [16, 18]. Anatomical location of Axial and Coronal T1 and T2, and contrast enhanced T-1 weighted MR images at initial diagnosis and at relapse were determined. Anatomical sites of disease were categorized as involving the CNS, ocular or extra-CNS. CNS lesions were further divided into the following sites: (a) right or left cerebral hemispheres with further classification into frontal, temporal or occipital lobes based on neuroanatomical land marks, (b) lesions involving the corpus callosum, (c) infra-tentorial posterior fossa lesions including the brainstem and cerebellum, (d) subependymal, (e) spinal cord or (f) leptomeningeal, based on the predominant location of the enhancing lesion. At relapse, enhancing lesions inside or within a 2 cm margin of the T2 hyperintensity at initial presentation was considered to be local relapse, while those outside this margin were considered distant relapse. Since PCNSL is a diffusely infiltrative disease with no clear margins, this 2 cm margin was chosen based on the margins used for radiation planning and pattern of relapse seen in glioblastoma [19]. Clinical history, including pretreatment Karnofsky Performance Score (KPS), cranial MRI with and without contrast, and computed tomography (CT) staging (chest and abdomen) scans were reviewed. Contrast-enhanced cranial CT was used in some early cases when MRI was not readily available. Results of bone marrow biopsy and ophthalmologic examination were reviewed when available.

## Results

A total of 129 consecutive newly diagnosed PCNSL patients received first line HD-MTX/BBBD during the study period; one patient treated with WBRT and six patients treated with maintenance immunotherapy were excluded. Of the remaining 122 patients, CR was achieved in 78 (64%) during or at the end of treatment, and 44 (56%) subsequently progressed. Due to the retrospective nature of this study, imaging was not available to confirm location of new enhancement at progression in three patients; additional four had no measurable lesion at progression (clinical progression or by CSF analysis). These seven were excluded from further analysis. We describe the location of first relapses, defined as new measurable enhancing lesion on contrast enhanced MRI in the remaining 37 patients.

**Fig. 1:**
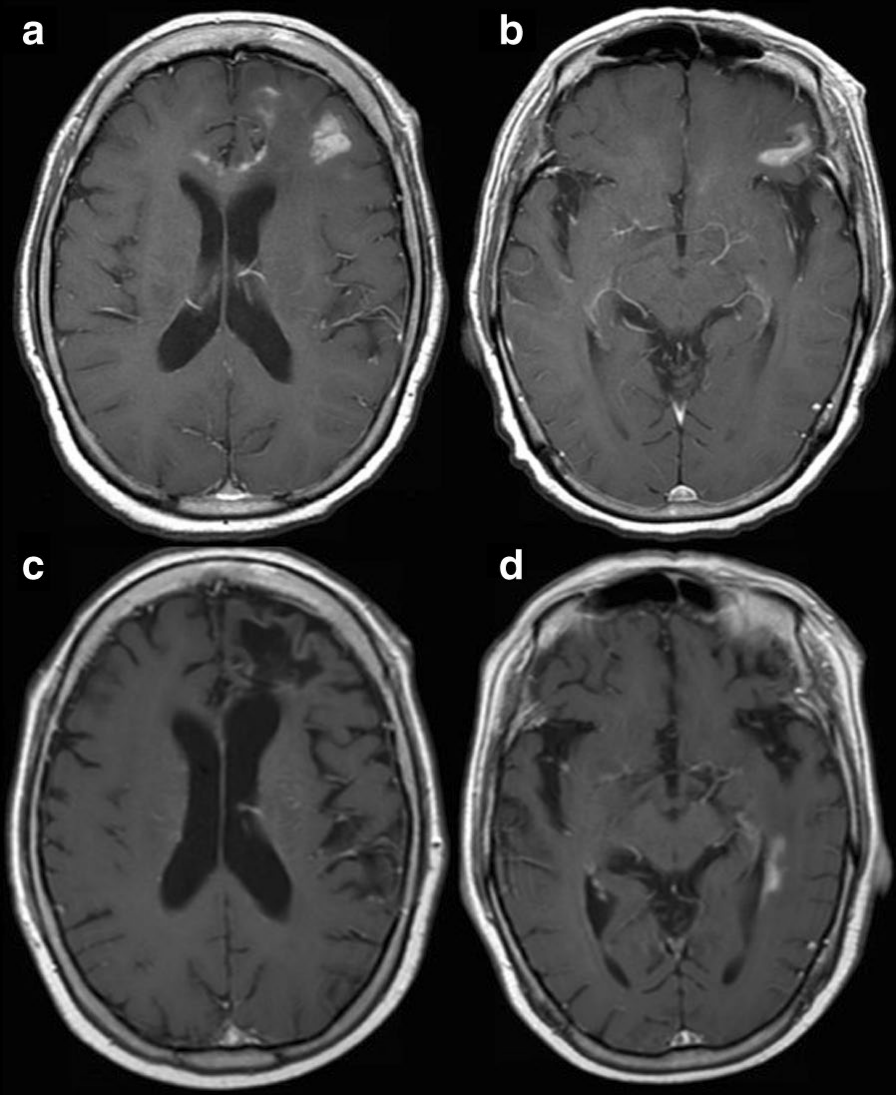
Case 1; Axial T1-weighted post contrast imaging at initial presentation (**a**, **b**), in complete response (**c**) after therapy and at relapse (**d**). At initial presentation, multiple scattered left frontal and callosal lesions are noted (**a**). Patient achieved complete response after 12 months of HD-MTX treatment (**c**). 1 year after finishing treatment, relapse was noted in the periventricular white matter of the left lateral ventricle (**d**)

**Fig. 2:**
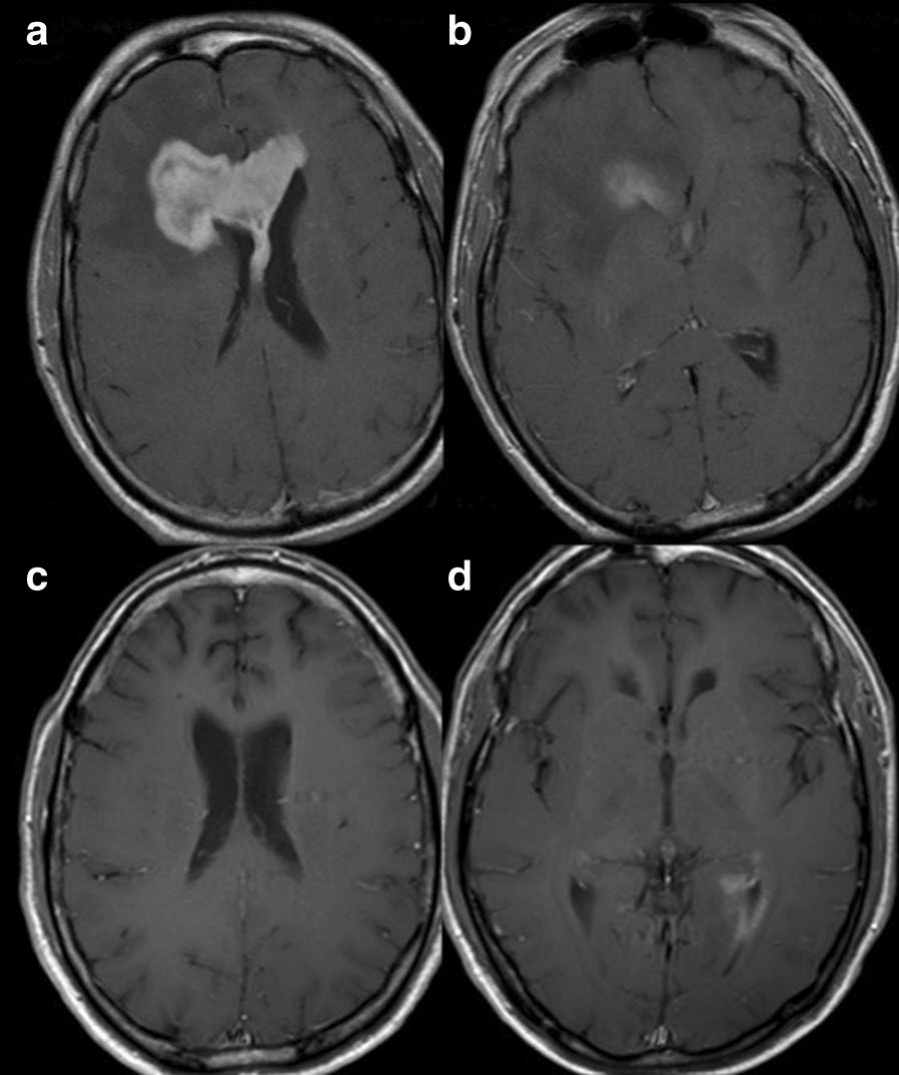
Case 2; Axial T1-weighted post contrast imaging at initial presentation (**a**, **b**) and relapse (**c**, **d**). Initial presentation shows a localized large enhancing lesion centered in the genus of the corpus callosum (CC) with extension mainly into the right frontal lobe (**a**, **b**). After 10 months of HD-MTX treatment there is relapse in the ependymal surface of the left lateral ventricle (**d**) with complete response in the CC lesion (**c**)

**Fig. 3:**
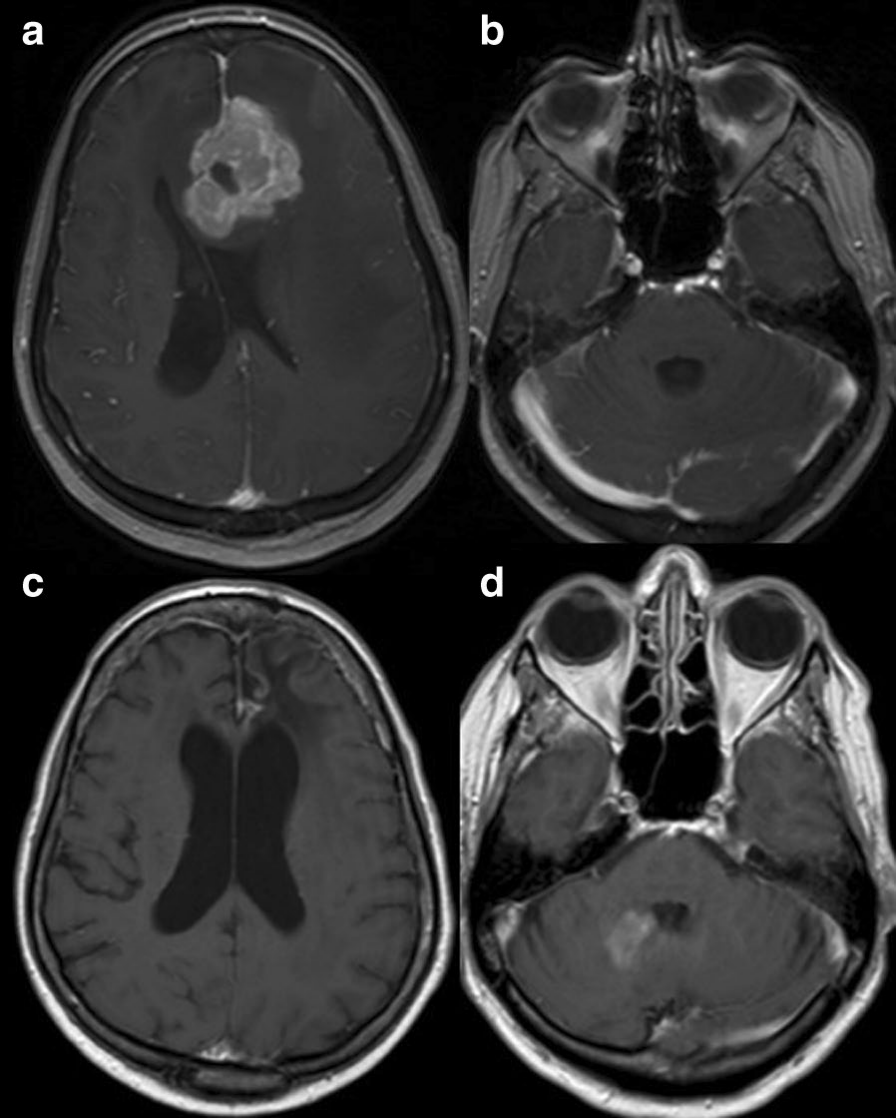
Case 3; Axial T1-weighted post contrast imaging at initial presentation (**a**, **b**), after completing therapy with HD-MTX (**c**) and relapse (**d**). Initial presentation shows a large enhancing lesion in the left frontal lobe (**a**) and normal cerebellum (**b**). Patient was in complete response after completing 1 year of HD-MTX treatment (**c**) with radiographic relapse in the right cerebellum, 6 years after the initial diagnosis (**d**)

**Fig. 4:**
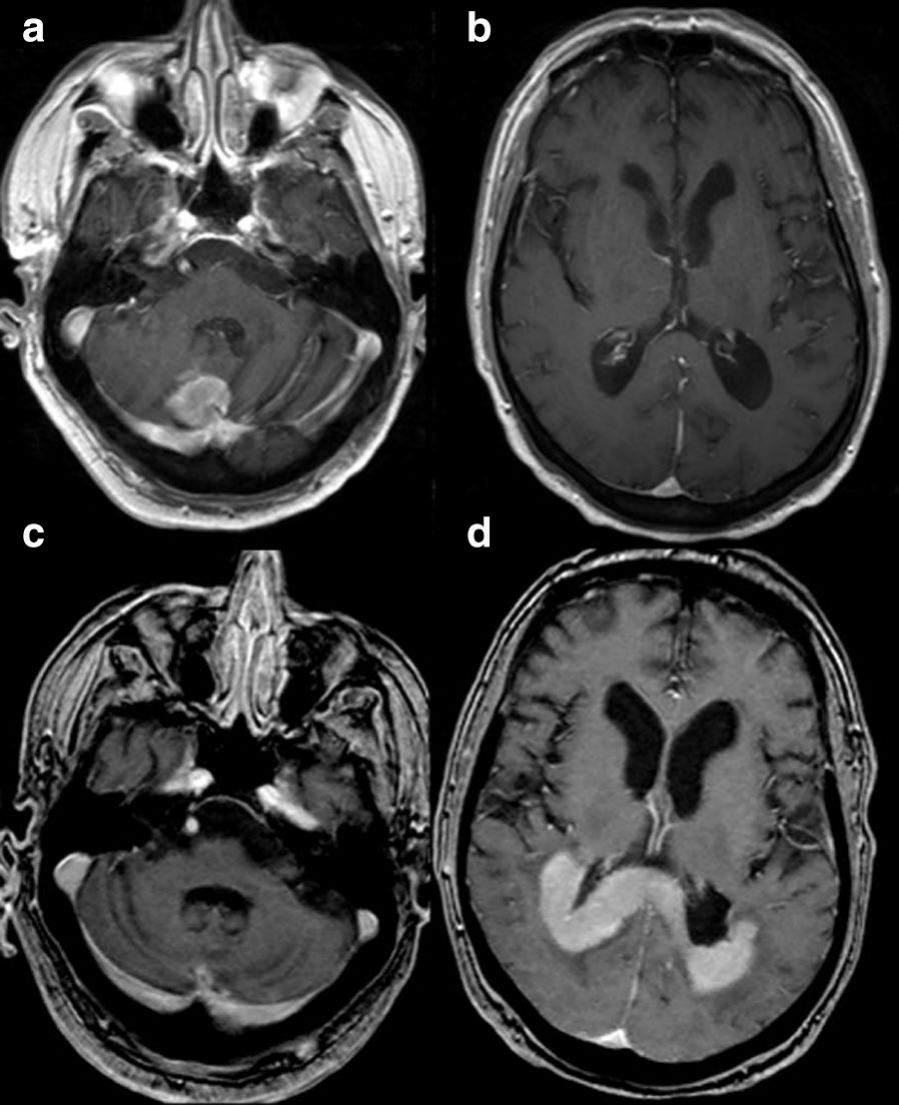
Case 4; Axial T1 WI post contrast imaging at initial presentation (**a**, **b**) and relapse (**c**, **d**). At initial presentation there is a solitary right cerebellar lesion (**a**) with normal supra-tentorial compartment (**b**). After 10 months of high-dose MTX treatment, relapse was noted in the splenium of the CC with extension to the periventricular white matter (**d**) with no evidence of residual disease in the cerebellum (**c**)

Among the 37 patients, ocular involvement at initial diagnosis was noted in seven patients. Bone marrow biopsy was performed in 29/37 patients, one patient had evidence of low grade lymphoma, and another had atypical polyclonal lymphoid aggregates, the remaining did not have any clinical evidence of lymphoma in the marrow. Demographics are described in Table 1. Median time to progression was 17.8 months (95% CI 11.7–41.7). At relapse, new enhancement was noted in a spatially distinct site in 30 of 37 (81%) patients with 15 (50%) relapses in an entirely different lobe. Six patients (20%) had systemic relapse (outside the CNS) with no new measurable lesion in the CNS (Table 2). Only 7/37 (19%) of relapses were contiguous (inside or within a 2 cm margin of T2 hyperintensity of at initial diagnosis; Table 2). Local relapses were more common when the initial lesion involved the corpus callosum, posterior fossa, sub-ependymal disease or the leptomeninges. Due to the small sample size, no formal statistical evaluation was performed. Representative cases are described in Figs. 1, 2, 3 and 4.

**Table 1:** Demographics of patients with relapsing primary central nervous system lymphoma

Number: 37	
Variables	
Median age in years at diagnosis (Min, Max)	63.7 (27.6, 80.1)
Gender (N, % Female)	21 (57%)
Median KPS at diagnosis (Min, Max)	80 (20, 100)
Ocular involvement (%)	7 (18.9%)
Treatment regiment	HD-MTX/BBBD without rituximab: 30 (81.1%)
	HD-MTX/BBBD with rituximab: 7 (18.9%)
Median age in years at relapse (Min, Max)	65.1 (31.6, 80.8)
Median time to progression in months	17.8 (95% CI 11.7–41.7)

*KPS* Karnofsky Performance status [55], *HD-MTX/BBBD* High dose methotrexate with blood–brain barrier disruption

**Table 2:** Site of relapse in primary central nervous system lymphoma

Site of relapse after complete response (n = 37)	
Distant	30 (81%)
Different lobe	15
Systemic/non CNS	6
Ocular	2
Same lobe but distinct site	3
Corpus callosum	2
Leptomeningeal	2
Local	7 (19%)
Corpus callosum	3
Cerebellum/posterior fossa	2
Sub-ependymal	1
Leptomeningeal	1

The location of enhancement at relapse after complete response with high dose methotrexate was at an anatomically distant site compared to the site of enhancement at location of enhancement at presentation in a majority of cases. Local relapses were more frequent when the initial lesion was in the corpus callosum, posterior fossa, subependymal or in leptomeningeal lesions

## Discussion

Both glioma and lymphoma are well recognized to involve the whole brain even when imaging may deceivingly suggest anatomically localized disease sites. Our data suggests that unlike gliomas where local relapse is the norm, the majority of PCNSL relapses occur distal to the site of initial presentation. This difference in the pattern of relapse may allow an insight into the mechanism of relapse. Our data aligns with a smaller retrospective study (16 patients) that evaluated relapse pattern in 16 PCNSL patients where only four relapses were at the site of initial tumor [20]. This study provides a larger sample size and duration of follow-up for this rare disease.

HD-MTX based therapy is the backbone of most modern chemotherapy regimens for PCNSL. The majority of PCNSLs are CD20-expressing diffuse large B cell lymphomas; the addition of rituximab, a monoclonal antibody (mAb) targeted against CD-20, to HD-MTX-based regimens is intuitive. However, there is ongoing debate regarding the delivery of high molecular weight monoclonal antibodies across the blood–brain barrier (BBB) and neurovascular unit (NVU) [21]. Monoclonal antibodies to CD-20 have a long half-life, and in pre-clinical studies have been shown to leak slowly and accumulate across diseased NVU around the enhancing tumor [22, 23]. Further, the addition of anti-CD20 mAb, rituximab to HD-MTX has substantially improved CR rates, progression free survival and OS in PCNSL [24, 25].

A similar pattern of distant relapses has also been reported in systemic DLBCL, where most relapses are believed to be derived from the same clones as the original tumor [14]. Similarly, small studies have suggested clonal relation of the primary and recurrent tumors in PCNSL [6, 15]. The possibility of late relapses and isolated CNS relapses of systemic lymphoma being new clones or the possibility of dual clonality has also been previously raised based on small studies [26, 27]. Although limited, there is emerging evidence suggesting that relapses after intravenous HD-MTX at sites distant from initial presentation within the CNS and in rare instances outside the CNS are not uncommon [20, 27–30]. The incidence and pattern of relapses distant from the site of initial tumor presentation with HD-MTX/BBBD (30/37, 81%) are comparable to those described with intravenous HD-MTX (12/16, 75%) [20]. These studies suggests that the pattern of distant relapse is unique to the natural history of PCNSL and unlikely related to BBBD.

The mechanism of relapse is unclear, but two mechanisms have been postulated (1) seeding from occult reservoir lesions within the CNS (including eye and CSF) or (2) seeding from the blood and bone marrow [7, 22, 31]. The first possibility is supported by preclinical studies that have demonstrated dormant microscopic PCNSL cells behind a minimally leaky NVU [22]. New contrast enhancement detected by MRI is generally considered to be a sensitive biomarker for disease progression. However, contrast enhancement is a sensitive marker for disrupted NVU which consists of the BBB (endothelial cells and their associated tight junctions) surrounded by neurons and non-neuronal cells such as pericyte and glial (astrocytes, microglia and oligodendroglia) foot processes [32–34]. Subclinical CNS tumor sites may remain undetected by conventional contrast enhanced MRI scanning behind an intact NVU or blood-cerebrospinal fluid barrier. At relapse, 10% (4/36) of our patients had radiographic evidence of leptomeningeal relapses; this raises the issue of occult lymphoma cells in the CSF reservoirs that subsequently seed sites distant from the initial tumor [1]. We acknowledge that contrast enhanced MRI may be an inadequate tool to detect microscopic (occult) disease. The low sensitivity of CSF cytology and the retrospective nature of this study further limits extensive review of CSF as an occult reservoir. However, prior studies looking at this issue with CSF-flow cytometry and CSF-polymerase chain reaction (PCR) found similar low rates (11–16%) of CSF dissemination of PCNSL [35]. On the other hand, the detection of persistent monoclonal B cells in blood and bone marrow samples by PCR in patients with no other evidence of systemic involvement by routine staging procedures supports the notion of seeding from these sites [31]. Unusually high mutation frequency of *Ig* genes seen at relapse also suggests that these clones are derived from these occult sites with ongoing mutation [36, 37].

Although this study is limited by its retrospective nature and small sample size, our report is an independent confirmation of the pattern of relapses in a larger cohort of patients treated with first line HD-MTX/BBBD. At relapse, tumor biopsy and bone marrow biopsy samples are not routinely collected in PCNSL patients; these additional assays would further help in establishing the mechanism and source of relapse in this rare disease. There is emerging evidence and interest in evaluating the role of blood- and CSF-based biomarkers such as circulating tumor cells (CTC), as well as micro-RNA and DNA in the blood and CSF to improve the diagnostic sensitivity and specificity [38–41]. Although there is limited data in PCNSL, CTC have been detected in a variety of solid tumors, including systemic DLBCL, lung, breast and prostate cancer; they may help with effective surveillance and early detection of persistent clonal populations even after radiographic CR as well as potentially help modify therapy, based on response [40, 42, 43].

The role of the BBB and NVU need to be considered when developing strategies and approaches for new therapies and biomarkers for response assessment in PCNSL. Strategies aimed at delaying relapses including those that address seeding from occult reservoir lesions within the CNS (including the CSF and eye) and/or seeding from outside the CNS (blood and bone marrow), are currently being evaluated. WBRT after CR addresses the issue of occult CNS disease. However, WBRT is associated with significant concerns for neurocognitive toxicities [44, 45]. The efficacy and improved toxicity profile of reduced-dose radiotherapy after CR is an area of active investigation [46]. Intrathecal route of drug administration is an alternative approach to target the CSF reservoir [21, 47]. However, limited drug delivery into the brain parenchyma beyond the superficial (2–3 mm) around the subarachnoid space is expected due to the inherent interstitial fluid pressure in the brain. In spite of good cytological responses, the majority had disease progression within a year in a phase I trial evaluating intrathecal rituximab at relapse [23, 47, 48]. This approach needs further evaluation; however there is concern for increased neuro-toxicities, especially with intrathecal administration of drug combinations [49, 50]. On the other hand, myeloablative chemotherapy with autologous stem cell transplant, and systemic maintenance anti-CD20 immunotherapy, address the issue of seeding from extra CNS sites [7, 51–53]. Encouraging results were noted after autologous stem cell transplantation in patients who have attained CR, but this approach is associated with significant hematological and non-hematological grade-3 and 4 toxicities and up to 10% treatment related mortality [53, 54]. Maintenance immunotherapy with anti CD-20 antibody via systemic (iv) infusion is another approach that has been shown to be relatively safe and potentially beneficial in a preliminary retrospective review, most likely due to its action on subclinical disease sites outside the CNS and potentially on occult CNS sites [7, 51]. These antibodies can potentially address occult extra-CNS as well as CNS reservoir sites due to slow leak and accumulation across minimally disrupted BBB. This approach is being evaluated in a prospective multicenter randomized trial [7]. Currently available clinical imaging techniques and blood or CSF biomarkers cannot detect these occult disease sites behind an intact NVU or at other potential non-CNS sites, innovative strategies for early detection and targeting therapies to address these sites can further improve outcomes.

## Conclusion

Both gliomas and PCNSL are well recognized to be a whole brain disease, even if imaging may suggest anatomically localized lesions at presentation. It is important to recognize that contrast enhancement is a surrogate for BBBD and not for the true extent of tumor in the CNS. Our results suggest that unlike gliomas, majority of PCNSL recur at spatially distinct anatomical locations within, and in few cases, outside the CNS. This may either be due to seeding from occult lesions (areas of CNS with intact BBB or ocular) or other extra CNS sites such as the bone marrow. Recognizing patterns of relapse is key for early detection and may provide insight into potential mechanisms of relapse as well as help develop strategies to extend duration of complete response.

## Abbreviations

PCNSL: primary central nervous system lymphoma
CC: corpus callosum
CNS: central nervous system
CR: complete response
CTC: circulating tumor cells
DLBCL: diffuse large B-cell lymphoma
BBB: blood-brain barrier
BBBD: blood–brain barrier disruption
HD-MTX: high dose methotrexate
MRI: magnetic resonance imaging
NVU: neurovascular unit
WBRT: whole brain radiotherapy
